# Prepyloric gastric diverticulum case report: A rare anatomic abnormality with limited clinical consequences

**DOI:** 10.1016/j.amsu.2022.103288

**Published:** 2022-01-26

**Authors:** Connor Shea, Ann Sheets, Faiz Tuma

**Affiliations:** Central Michigan University College of Medicine, Department of Surgery, USA

**Keywords:** Diverticulum, Gastric diverticulum, Rare diverticulum, Prepyloric diverticulum, Asymptomatic diverticulum, Esophagogastroduodenoscopy, Case report

## Abstract

**Introduction:**

and Importance: Gastric diverticula (GDs) are typically formed on the posterior wall due to congenital or acquired causes. Although diverticula are not uncommon throughout the gastrointestinal tract, GDs are the least common type, and their presence in the prepyloric area is extremely rare. GDs are frequently asymptomatic but can present with serious complications that require surgical intervention in rare cases.

**Case presentation:**

A 54-year-old woman with a history of morbid obesity, hyperlipidemia, and diabetes mellitus (DM) presents with acute onset left upper quadrant (LUQ) abdominal pain. Based on presenting symptoms, an esophagogastroduodenoscopy (EGD) was performed to evaluate possible causes of abdominal pain. Interestingly, EGD revealed a moderately sized (3 cm) prepyloric diverticulum with a small polyp surrounded by normal gastric tissue. Biopsying of the intra-diverticular polyp revealed no abnormal pathology. Further assessment with Computerized Tomographic (CT) scan identified the diverticulum but with no other related gastric or gastrointestinal changes or pathology.

**Clinical discussion:**

The diverticulum was excluded as a cause of the pain. Hence, a conservative management approach was followed with no change in status for the following three months of observation. The patient continued to report non-specific symptoms but denied further episodes of abdominal pain or serious symptoms.

**Conclusions:**

GDs present with a wide variety of symptoms making the diagnosis difficult without thoroughly examining the entire anatomic region of potential pathology. Although GDs are rare, they are easily identified via EGD or imaging modalities. However, long-term follow-up information is needed to understand this clinical entity's behavior fully.

## Highlights

• Gastric diverticula in the pre-pyloric area the rarest kind.
• Pre-pyloric gastric diverticula are often asymptomatic but may present with a variety of symptoms including severe symptoms.
• Gastric diverticula are often discovered incidentally on esophagogastroduodenoscopy.
• Conservative management and diet modification are commonly used methods in the management of gastric diverticula.
• It is important to document gastric diverticula in order to analyze trends of incidence in the population.

## 1 Introduction

Gastric diverticula (GDs) are outpouchings of the stomach that most frequently form along the posterior wall [1]. They usually present as single lesions that range from 1 to 5 cm in diameter, with occasional reports describing lesions up to 11 cm^2^. Compared to other gastrointestinal tracts (GIT) diverticula, GDs are the least common form of diverticula. They are observed in 0.03–0.1% of upper gastrointestinal contrast studies, 0.03–0.3% of autopsy reports, and 0.01–0.11% of oesophagogastroduodenal endoscopies [1]. As it is evident from possible associated pathology, the presentation may vary. Hence, examining the entire anatomic region and all potential sources of symptoms is crucial to accurately identify the pathology of concern and the role of GD in symptomatology. This case report has been reported in line with the SCARE criteria [2].

Due to the rarity of this anatomic variation, studying GDs relies mainly on the case reports and series to understand this anatomic entity better. With the variation in presentation, size, and location, more information can be utilized to describe the clinical varieties and available management approaches. We report a case report of a middle-aged woman with a GD finding in the prepyloric area identified incidentally on the esophagogastroduodenoscopy (EGD) exam. In addition, we provide a literature review and discussion of the various presentations and management options.

## 2 Case report

**Fig. 1:**
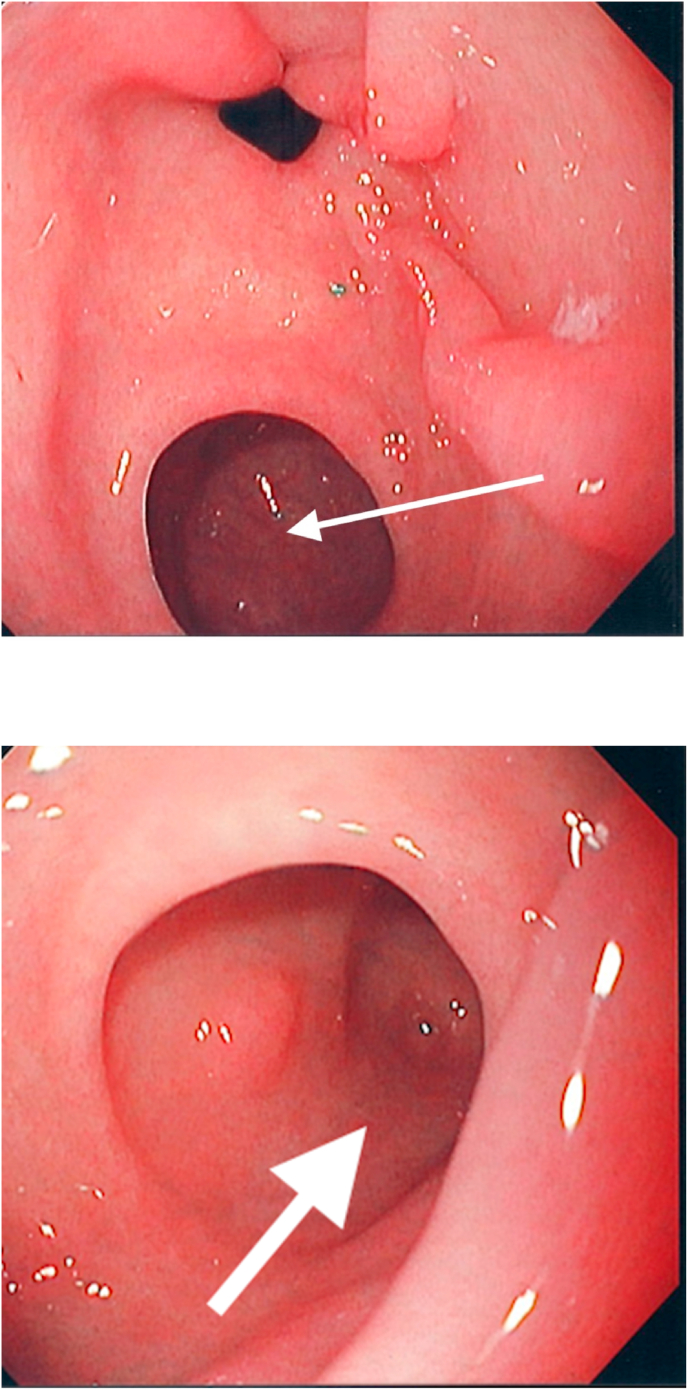
Endoscopic view of the prepyloric diverticulum.

A 54-year-old woman with a history of morbid obesity, hyperlipidemia, and diabetes mellitus (DM) presents with episodes of the left upper quadrant (LUQ) abdominal pain of two months duration. Other than occasional heartburn and bloating, she denied other significant symptoms of upper GIT. A recent ultrasound examination revealed a Gall Bladder polyp. Three months earlier, she was diagnosed with COVID-19 and was hospitalized with pneumonia. At presentation, her physical examination revealed reproducible tenderness in the LUQ. The treating team performed an EGD exam. The EGD revealed a moderate size prepyloric diverticulum on the lesser curvature side (Fig. 1).

**Fig. 2:**
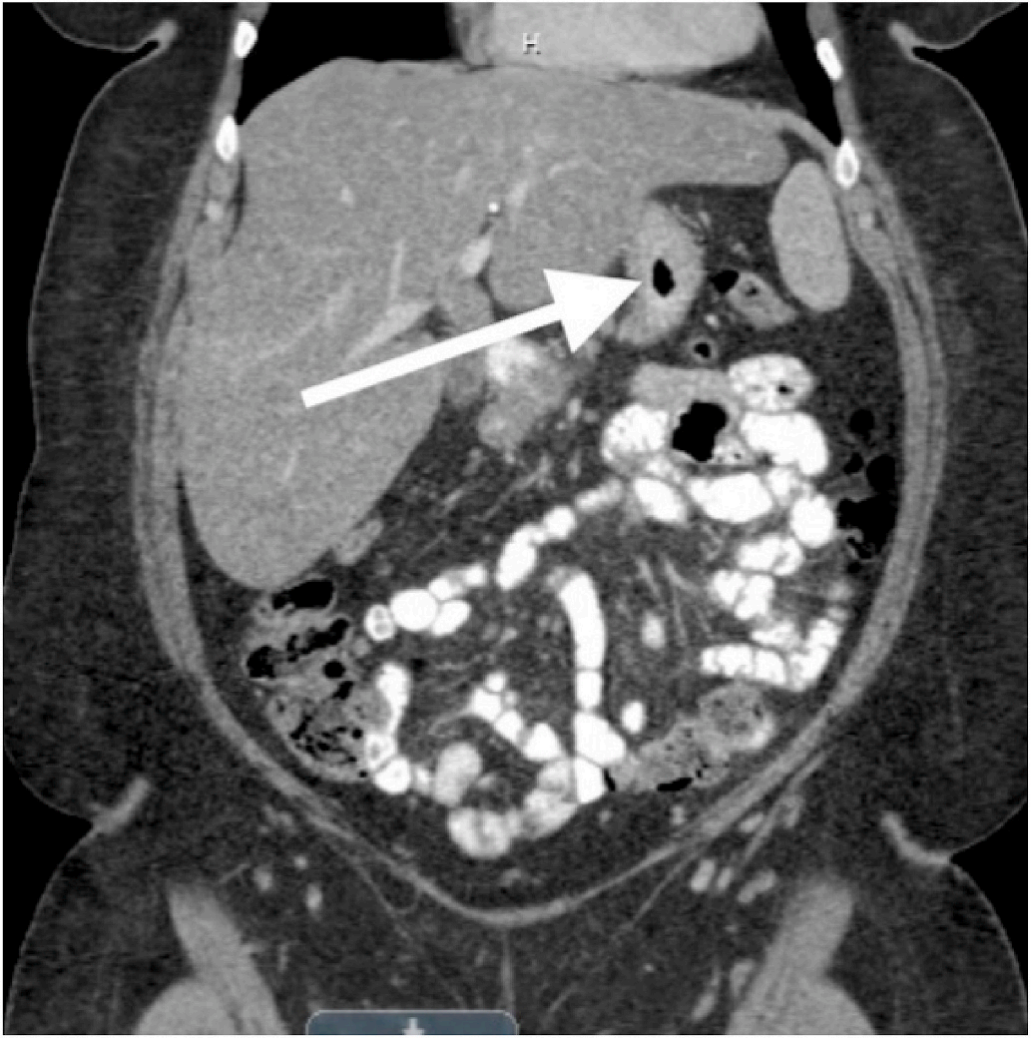
Computerized Tomography of the prepyloric diverticulum.

Interestingly, a small polyp was identified inside the diverticulum surrounded by normal gastric tissue. Biopsy of the polyp tissue revealed normal gastric mucosa. Further assessment of the patient with Computerized Tomography (CT) showed the diverticulum (Fig. 2) with no surrounding pathology or complications. No other gastrointestinal (GI) related abnormalities were identified in the CT. Findings were discussed with the patient. The diverticulum is not found to be a source of symptoms. Observation and conservative management were proposed. The patient did have any new symptoms or changes in the status for three months after the diagnosis. She continued to report non-specific symptoms of occasional bloating with ingestion but denied further abdominal pain episodes or other significant symptoms.

## 3 Discussion

Gastric diverticula are rare findings that are usually identified incidentally during EGD examination. Pre-pyloric antral diverticula, such as the case we present, is even rarer than the average GD. Two types of GDs generally occur in two different locations of the gastric wall. Congenital GDs comprise 72% of all GDs, and they are commonly found within 2–3 cm of the gastroesophageal junction along the posterior wall of the stomach. Congenital GDs are considered “true” diverticula because they contain all layers of the GI wall (mucosa, muscularis propria, and adventitia) [3]. Congenital GDs are caused by a defect in the gastric wall musculature that results in the lack of longitudinal muscle fibers leaving only circular muscle fibers present in the gastric wall. This defect results in weak areas through which a diverticulum can develop during the fetal period [3]. Congenital GDs are found in conjunction with other diverticula, peptic ulcer disease, pancreatitis, hepatitis, and cholecystitis [4,[3], [5], [6]].

Acquired GDs are less common than congenital GDs, and they generally occur near the antrum. They are considered pseudodiverticula or “false” diverticula because they do not include all three gastric wall layers (only contain the mucosa and submucosal layers) [7]. Hence, further assessment is required to identify and characterize the type and potential consequences of the diverticula. Acquired GDs are associated with peptic ulcer disease, pancreatitis, cholecystitis, malignancies, and gastric outlet obstructions [7]. Even though the diverticulum was identified in the antrum, the absence of the prior history of gastric disease and the nature of the diverticulum (true) makes it unlikely to be acquired GD.

GDs are equally distributed between men and women between the ages of 20–60 years old, with only 4% of GDs occurring in patients under 20 [7]. The majority of diverticula are found on the posterior wall of the cardia region and along the lesser curvature of the stomach. The presence of GDs in the prepyloric area is extremely rare [8]. This rarity increases the scientific value of discussing and reporting these cases to foster our knowledge about this type of GD.

GDs are often identified using radiological examinations using upper gastrointestinal contrast radiographic studies or abdominal CT scanning with oral contrast administration. Upper gastrointestinal endoscopic examinations also identify GDs, often incidentally [8]. Most GDs are asymptomatic, probably because most are “true” diverticula and not pseudodiverticula. The case we are presenting is a classic example of this category. Symptoms, if present, typically manifest as a vague sensation of fullness or discomfort in the upper abdomen, with the most common symptom being abdominal pain (present in 18–30% of cases) [9,10]. Other symptoms include epigastric pain, nausea, vomiting, dyspepsia, early satiety, and vague dysphagia [1,10]. Presenting symptoms may also be associated with major complications of GD, such as acute upper gastrointestinal bleed or perforation, which require immediate surgical attention [1]. It has been hypothesized that the diameter of the diverticulum may correlate with the development of symptoms. Wider openings are usually less symptomatic, possibly because food and other gastric enzymes are less easily trapped than diverticula with more narrow openings [11].

It is well documented that asymptomatic GD's do not require specific treatment. Conservative management is the preferred course of action following the diagnosis of an asymptomatic diverticulum. In cases without serious complications, the use of proton pump inhibitors and a soft diet may alleviate mild symptoms [7].

Around 10% of patients with true GD's require surgical intervention. Unfortunately, these cases often present with serious complications such as bleeding, perforation, or ulceration. Other indications for surgery include cases that are not resolved using conservative management. The optimal surgical procedure is resection of the GD with primary repair [12]. Laparoscopic resection has been shown to be a feasible, safe technique with an excellent outcome and is the primary treatment when GDs are symptomatic. Surgical resection of GDs is a relatively simple procedure that can be performed both openly or laparoscopically, but the latter is currently the preferred method compared to the former. However, resection of the incorrect part of the stomach has been reported [13]. Therefore it is recommended that the procedure be combined with intraoperative endoscopy. Endoscopy would allow identifying elusive GDs by stretching the diverticular sac [14].

It is essential to study this clinical finding further and investigate the potential correlations with symptoms and complications. Practitioners are encouraged to report GDs to improve our understanding of their behavior. Further studies and attention to this clinical finding should be considered to document the incidence of this rare abnormality and analyze trends of prevalence in the population. Due to the lack of data in the field, the prevalence and association of GDs with symptoms or complications are currently unknown. With a better understanding of the behavior and trend of GDs, practitioners are more informed to manage GDs.

## 4 Conclusions

GD is a rare entity that is usually identified incidentally during EGD examination. Therefore, adequate assessment of the upper GI region is indicated to characterize the finding further and assess potentially associated abnormalities. Most GDs are asymptomatic and require no active management. However, significantly symptomatic or complicated GDs may benefit from surgical excision.

## Conflicts of interest, source of funding, IRB, and informed consent

The authors report no conflicts of interest and no funding. IRB was not consulted informed consent obtained.

## Provenance and peer review

Not commissioned, externally peer-reviewed.

## Sources of funding

N/a.

## Ethical approval

Our institution does not require ethical approval for reporting individual cases or case series (Central Michigan University Medical Education Partners), N/a.

## Consent

Written informed consent was obtained from the patient to publish this case report and accompanying images. A copy of the written consent is available for review by the Editor-in-Chief of this journal on request.

## Author contributions

Connor Shea, MS^2^: Writing the draft, data collection, editing, submitting, revising.

Ann Sheets, RN: Data collection, documentation gathering.

Faiz Tuma, MD, MME, MDE, EdS, FACS, FRCSC: Conceptualizing, writing, editing, supervising.

## Research registration

N/a.

## Guarantor

Faiz Tuma, MD, MME, MDE, EdS, FACS, FRCSC.

## Declaration of competing interest

None declared.
